# Hybridizing Fabrications of Gd-CeO_2_ Thin Films Prepared by EPD and SILAR-A+ for Solid Electrolytes

**DOI:** 10.3390/molecules30030456

**Published:** 2025-01-21

**Authors:** Taeyoon Kim, Yun Bin Kim, Sungjun Yang, Sangmoon Park

**Affiliations:** 1 Department of Engineering in Energy Materials, Graduate School, Silla University, Busan 46958, Republic of Korea; 2 UNIST Central Research Facilities, Ulsan National Institute of Science and Technology, Ulsan 44919, Republic of Korea; 3 Department of Environmental Energy & Chemistry, College of Engineering, Silla University, Busan 46958, Republic of Korea; 4 Department of Fire Protection and Safety Management, College of Health and Welfare, Silla University, Busan 46958, Republic of Korea

**Keywords:** gadolinium doped ceria, thin film, SILAR-A+, EPD, hydrothermal annealing

## Abstract

Thin films of gadolinium-doped ceria (GDC) nanoparticles were fabricated as electrolytes for low-temperature solid oxide fuel cells (SOFCs) by combining electrophoretic deposition (EPD) and the successive ionic layer adsorption and reaction-air spray plus (SILAR-A+) method. The Ce_1−*x*_Gd*_x_*O_2−*x*/2_ solid solution was synthesized using hydrothermal (HY) and solid-state (SS) procedures to produce high-quality GDC nanoparticles suitable for EPD fabrication. The crystalline structure, cell parameters, and phases of the GDC products were analyzed using X-ray diffraction. Variations in oxygen vacancy concentrations in the GDC samples were achieved through the two synthetic methods. The ionic conductivities of pressed pellets from the HY, SS, and commercial G_0.2_DC samples, measured at 150 °C, were 0.6 × 10^−6^, 2.6 × 10^−6^, and 2.9 × 10^−6^ S/cm, respectively. These values were determined using electrochemical impedance spectroscopy (EIS) with a simplified equivalent circuit method. The morphologies of G_0.2_DC thin films prepared via EPD and SILAR-A+ processes were characterized, with particular attention to surface cracking. Crack-free GDC thin films, approximately 730–1200 nm thick, were successfully fabricated on conductive substrates through the hybridization of EPD and SILAR-A+, followed by hydrothermal annealing. EIS and ionic conductivity (1.39 × 10^−9^ S/cm) measurements of the G_0.2_DC thin films with thicknesses of 733 nm were performed at 300 °C.

## 1. Introduction

**Figure 1:**
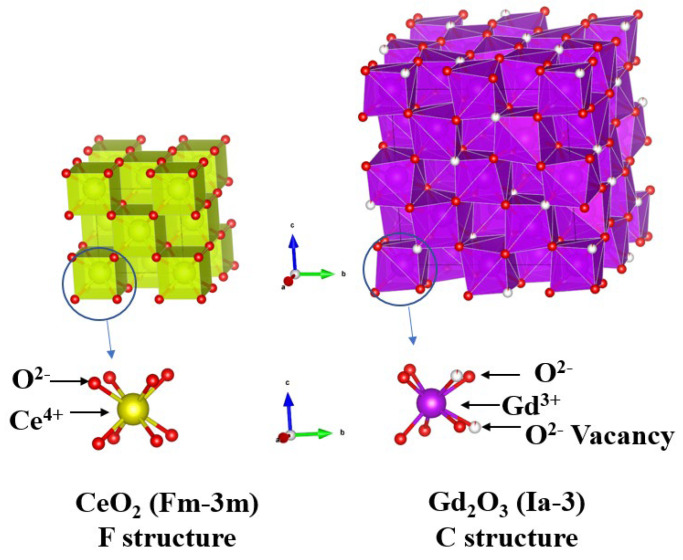
The structures of CeO_2_ (F-structure, Fm-3m) and Gd_2_O_3_ (C-structure, Ia-3).

Since the first investigation of a solid electrolyte for solid oxide fuel cells (SOFCs) in 1937, oxide-ion (O^2−^)-conducting materials fueled by hydrogen have been extensively studied as solid electrolytes in SOFCs due to their appropriate electrochemical reactions at working temperatures [1,2,3,4,5,6,7,8]. One of the primary challenges in developing suitable O^2−^-conducting solid electrolytes is overcoming the high operating temperature limitation to reduce maintenance costs and improve system stability. SOFCs are categorized into high (~1000 °C), intermediate (600–800 °C), and low (<600 °C) temperature ranges based on their operating conditions [1,2,3,4,5,6,7,8]. While yttrium-stabilized zirconia (YSZ) has demonstrated excellent O^2−^ conductivity along with thermal, chemical, and mechanical stability, its effectiveness is constrained to high operating temperatures (~1000 °C). Efforts to address this limitation have included fabricating ultrathin YSZ films via atomic layer deposition, which reduces operating temperatures by minimizing ohmic losses [9]. In parallel, intermediate-temperature electrolytes with superior O^2−^ conductivity have been developed, such as samarium-doped ceria (SDC) and gadolinium-doped ceria (GDC) [5,6,7,8]. The ceria (CeO_2_) structure, which enables oxygen conductivity through the formation of oxygen vacancies, adopts a fluorite-type (F-structure, Fm-3m) crystal lattice, as shown in Figure 1 [10,11]. When Sm^3+^ or Gd^3+^ ions substitute Ce^4+^ in the CeO_2_ lattice, the introduction of oxygen vacancies significantly enhances ionic conductivity [5,6,7,8]. However, as the concentration of Sm^3+^ or Gd^3+^ increases, the F-structure gradually transitions to a cubic C-structure (Ia-3) typical of Sm_2_O_3_ or Gd_2_O_3_, as depicted in Figure 1 [5,6,7,8,10,11]. Additionally, the Ce-O_8_ bonds in the F-structure transform into Ce(Gd,Sm)-O_6_ bonds with reduced coordination as Sm^3+^ or Gd^3+^ ions accumulate.

Thin films of these electrolytes have been prepared using techniques such as sputtering, spin-coating, pulsed laser deposition, and chemical vapor deposition [9,12,13,14,15,16,17]. In a previous study, crack-free YSZ thin films were achieved using the successive ionic layer adsorption and reaction-air spray plus (SILAR-A+) method, which integrates SILAR with air spray steps [18,19]. This method enables the formation of thin films composed of nanoparticles via dehydration and crystallization during post-hydrothermal annealing at temperatures below 200 °C. Repeated cycles of adsorption, rinsing, precipitation, and air spray deposition on oxidized substrates allow for the fabrication of ultrathin films. In this study, thin films of crystallized GDC nanoparticles were successfully fabricated using a hybrid deposition process combining electrophoretic deposition (EPD) with the SILAR-A+ method, followed by post-hydrothermal annealing. The structure, surface morphology, and ionic conductivity of the GDC films were characterized using X-ray diffraction, scanning electron microscopy, and electrochemical impedance spectroscopy, respectively.

## 2. Results and Discussion

**Figure 2:**
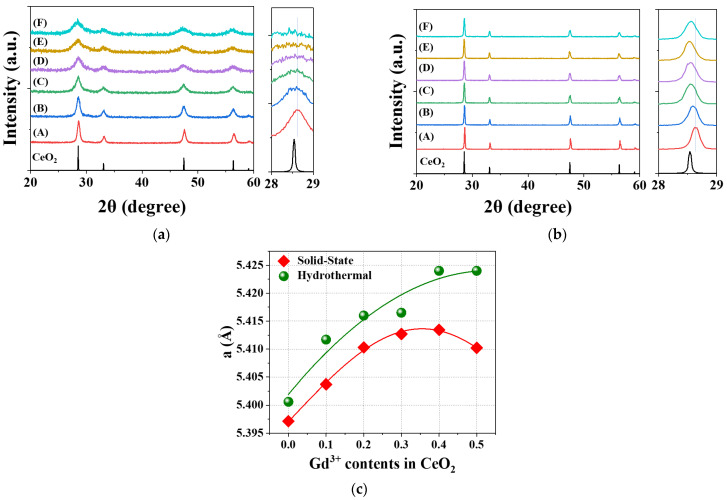
The calculated X-ray powder diffraction (XRD) patterns of cubic CeO_2_ (ICSD 28753) phase and the obtained XRD patterns of Ce_1−*x*_Gd*_x_*O_2−*x*/2_ (G*_x_*DC, *x* = 0(A), 0.1(B), 0.2(C), 0.3(D), 0.4(E), 0.5(F)) powders prepared by (**a**) hydrothermal and (**b**) solid-state methods and (**c**) the cell parameters as a function of Gd^3+^ contents.

Gd^3+^ ions were doped into the CeO_2_ host lattice (Ce_1−*x*_Gd*_x_*O_2−*x*/2_ (G*_x_*DC), *x* = 0.1–0.5) to prepare solid electrolytes for low-temperature solid oxide fuel cells (SOFCs) using hydrothermal (HY) and solid-state (SS) methods. As shown in Figure 2a,b, the gradual increase in Gd^3+^ ion content in CeO_2_ caused shifts in the X-ray diffraction (XRD) patterns to lower and higher angles, particularly in the Bragg reflection positions around 2θ = 28–29° for both HY and SS methods. The calculated lattice parameter (*a*) of GDC showed a nonlinear trend, initially increasing and then decreasing with the increase in Gd^3+^ ion content, as depicted in Figure 2c and Table S1. R_p_ values, indicating good agreement between the observed and calculated reflections, ranging from 6.9 to 11.1%, are observed in all samples prepared using both HY and SS methods. The *a* values reached their maximum at *x* = 3 for the SS method and *x* = 4 for the HY method. Interestingly, the unit cell of GDC synthesized via the SS method was significantly smaller than that prepared by the HY method. This difference is likely due to the formation of more oxygen vacancies in the SS GDC lattice, which are induced by the high-temperature conditions of the SS synthesis process.

**Figure 3:**
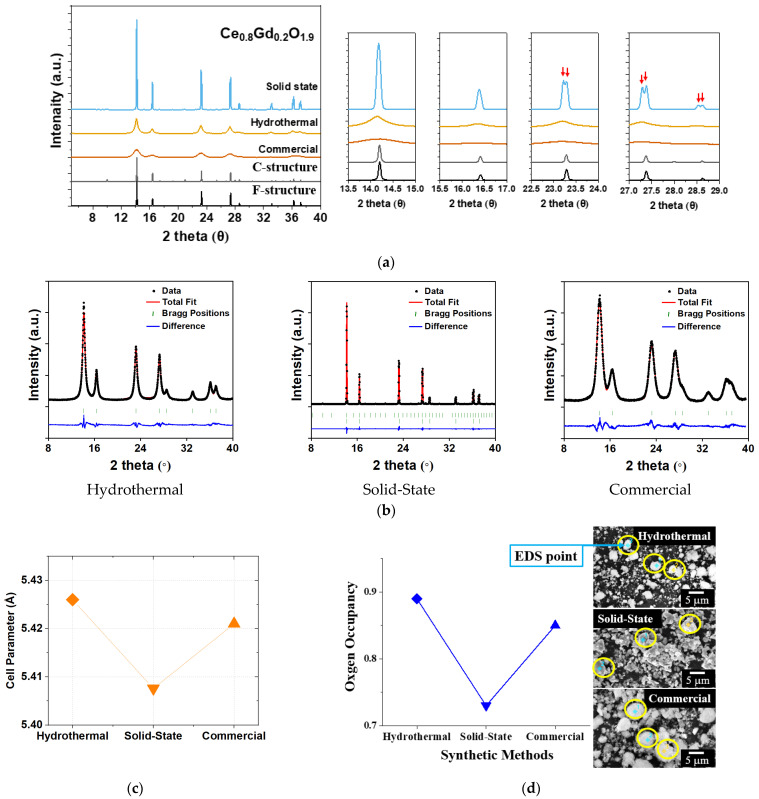
(**a**) The calculated XRD patterns of F- and C-structures and the obtained synchrotron XRD patterns and (**b**) structure refinements of Ce_0.8_Gd_0.2_O_1.9_ (**c**) cell parameters and (**d**) oxygen occupancy with SEM images vs. HY, SS, and commercial sources.

The synchrotron powder XRD data for the G_0.2_DC samples (Ce_0.8_Gd_0.2_O_1.9_; commercial, HY, SS) are shown in Figure 3a and Table S2. The full width at half maximum (FWHM) of the XRD peaks for both the commercial and HY samples is noticeably broader compared to that of the SS sample due to the particle sizes. R_p_ values below 4.44% were observed in all the G_0.2_DC samples. The chi-square (χ^2^) values of the refined G_0.2_DC samples represent goodness of fit, as shown in Table S2. The χ^2^ values for HY and commercial G_0.2_DC samples (7.16 and 10.5, respectively) are larger than that of the SS G_0.2_DC sample (0.979). Figure 3b presents the cell parameter (*a*) and oxygen occupancy, along with SEM images, for the HY, SS, and commercial G_0.2_DCs, respectively.

**Figure 4:**
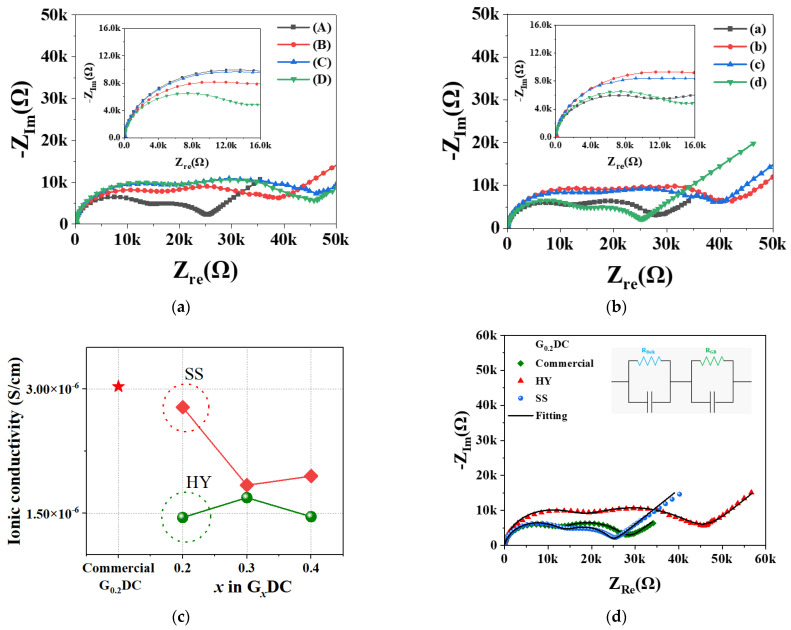
Electrochemical impedance spectroscopies (EISs) of (**a**) HY, (**b**) SS G*_x_*DC (commercial (A), *x* = 0.2(B), 0.3(C), 0.4(D)), (**c**) their ionic conductivities with commercial sources at 150 °C, and (**d**) EIS data of G_0.2_DC samples fitted via an equivalent circuit.

The *a* values and oxygen occupancies were derived from synchrotron powder XRD analysis and energy dispersive X-ray spectroscopy (EDS), respectively. The cell parameter and oxygen occupancy values for HY and commercial G_0.2_DCs were similar, measured as 5.426(2) Å and 5.421(3) Å, and 0.89 and 0.85, respectively. However, the SS G_0.2_DC sample exhibited significantly lower values, with *a* = 5.40761(4) Å and oxygen occupancy of 0.73. In the SS G_0.2_DC sample, peak splitting was observed in the XRD patterns at higher angular resolution (2θ), as shown in Figure 3a. This peak splitting suggests the coexistence of mixed phases: the F-structure of CeO_2_ and the C-structure of Gd_2_O_3_, resulting in distinct cell parameter and oxygen vacancy characteristics. For the GDC prepared using the HY method, the lattice parameter (*a*) as a function of Gd³^+^ ion content exhibited nonlinear behavior, as shown in Figure 2c. This trend may be attributed to a reduction in the average connectivity of Ce-O_8_; in the F-structure, driven by a decrease in the coordination number around Ce^4+^ cations, which generates oxygen anion vacancies. Electrochemical impedance spectroscopies (EISs) for pressed pellets of HY and SS G*_x_*DC (*x* = 0.2, 0.3, 0.4), and commercial G_0.2_DC powders are shown in Figure 4a,b, respectively. The total (σ_Total_), which is combination of bulk (σ_bulk_) and grain boundary (σ_gb_) contributions, conductivity was calculated from the EIS fits at 150 °C in Figure 4c, Figure S1 and Table S3.

The σ_Total_ values for HY and SS G*_x_*DC (*x* = 0.2, 0.3, 0.4) were 1.4 × 10^−6^, 1.7 × 10^−6^, 1.4 × 10^−6^ and 2.8 × 10^−6^, 1.8 × 10^−6^, 1.9 × 10^−6^ S/cm, respectively (Figure S1 and Table S3). In addition, the σ_Total_ for the commercial G_0.2_DC sample was 3.1 × 10^−6^ S/cm (Figure S1 and Table S3). Among the HY, SS, and commercial samples, the HY G_0.2_DC exhibited a relatively low σ_Total_ value, which may be attributed to its smaller grain size (Figure S2), resulting in enhanced interfacial resistance [20]. The EIS raw data for G_0.2_DC samples were analyzed using a simplified equivalent circuit model, comprising a capacitor, bulk resistance (R_Bulk_), and grain boundary resistance (R_GB_). The analysis was performed using the EIS Spectrum Analyzer, as shown in Figure 4d, Figure S1 and Table S3 [21,22,23]. The σ_Total_ values obtained from the fitting process for HY, SS, and commercial G_0.2_DC samples were 1.6 × 10^−6^, 2.6 × 10^−6^, and 2.9 × 10^−6^ S/cm, respectively (Figure S1 and Table S3). The ionic conductivity values derived from the raw and the fitted data exhibited differences in the range of 6–12%.

**Figure 5:**
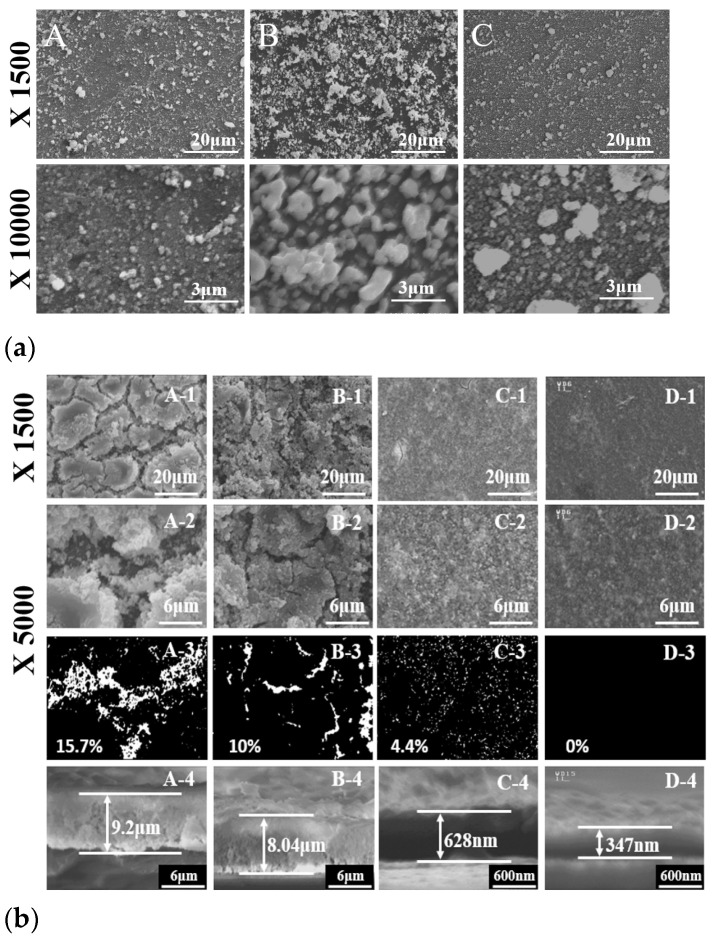
(**a**) Surface SEM images of the electrophoretic depositions (EPDs) of G_0.2_DC prepared by A. HY, B. SS, C. Commercial sources and (**b**) surface and cross-sectional SEM images of G_0.2_DC thin films by SILAR (A,B-1 to 4) and SILAR-A+ (C, D-1 to 4) methods (as-made (A, C) and subsequent hydrothermal annealing (B, D) at 175 °C).

The HY, SS, and commercial G_0.2_DC particles were deposited on ITO glass using the electrophoretic deposition (EPD) method. The deposition utilized a suspension containing 0.1 g of G_0.2_DC and 0.02 g of iodine in 50 mL of acetone, with a DC power supply applied at 20 V for 15 s. Figure 5a(A–C) shows SEM images of the surface morphologies for HY, SS, and commercial G_0.2_DCs, respectively. The particles were fully coated on the ITO substrate. HY G_0.2_DC particles were uniformly deposited, while the SEM images of SS and commercial G_0.2_DCs exhibited large grains and aggregation, respectively. Figure 5b illustrates G_0.2_DC thin films prepared using two methods: successive ionic layer adsorption and reaction (SILAR, A and B) and SILAR-air spray plus (SILAR-A+, C and D), with 150 cycles.

**Figure 6:**
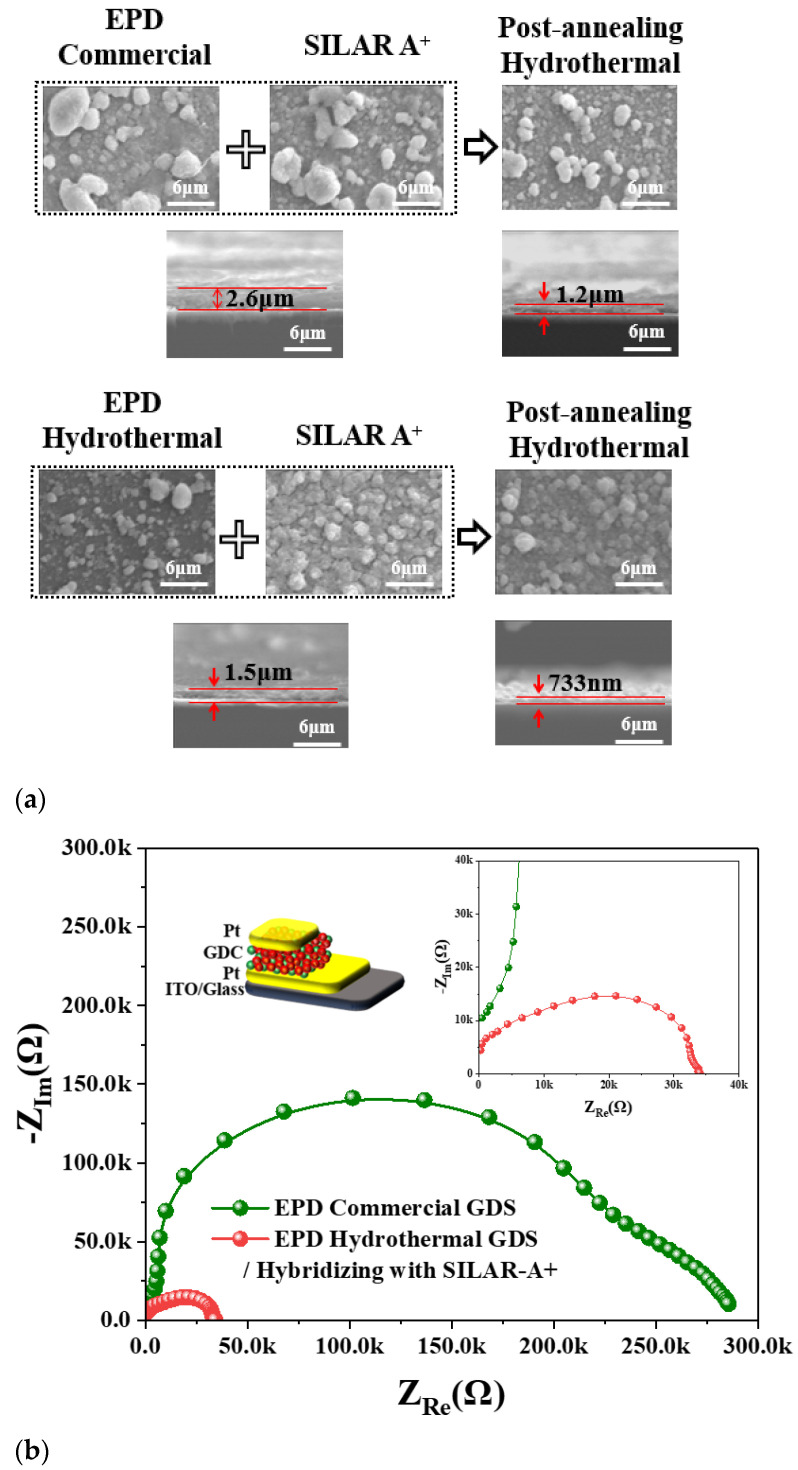
(**a**) SEM images and (**b**) EIS of G_0.2_DC thin films by hybridizing fabrications of EPD (commercial, HY) and SILAR-A+ process.

During each cycle of the SILAR process, the glass substrate was sequentially immersed in a Gd^3+^/Ce^4+^ cation solution for adsorption, rinsed in water to remove spectator anions, transferred to an OH^−^ anion solution for precipitation, and then rinsed again to eliminate residual layers. SEM images in Figure 5(A–D) represent G_0.2_DC films without post-annealing (A, C) and with additional post-hydrothermal annealing at 175 °C (B, D). Each set of images (A, B, C, D-1 and D-2) was magnified by 1500× and 5000× to observe surface cracks. Figure 5 (A,B,C,D-3) shows crack and crack-free SEM images in white and black contrasts. In as-made films (A-3 and C-3), surface cracks were clearly reduced from 15.7% to 4.4%, and from 10% to 0% after annealing (B-3 and D-3), demonstrating the effectiveness of the SILAR-A+ process combined with post-hydrothermal annealing in producing crack-free G_0.2_DC films. Figure 5b (A,B,C,D-4) presents cross-sectional SEM images showing the thickness of G_0.2_DC thin films, accompanied by bar graphs. The thicknesses of films produced via the as-made SILAR and SILAR-A+ processes (A-4 and C-4) were 9.2 μm and 0.628 μm (628 nm), respectively, while those of post-hydrothermally annealed films (B-4 and D-4) were 8.04 μm and 0.347 μm (347 nm), respectively. Hydrothermal annealing removed crystallized hydroxyl and water layers, resulting in thinner cross-sections, as observed in SEM images. The reduced thickness and crack formation in the films were attributed to the efficient removal of diffusion layers during the air-spray process and the subsequent exclusion of hydroxyl and water layers through hydrothermal annealing at 175 °C. In a previous report, freestanding YSZ (Y-doped ZrO_2_) membranes with thicknesses ranging from 500 nm to 2 µm were fabricated to test power density at 600 °C [24]. Further advancements reduced the thickness of YSZ membranes to 50 nm, enabling SOFCs to operate at lower temperatures, around 350 °C [24]. Deposition techniques such as pulsed laser deposition, chemical vapor deposition, spin coating, and physical vapor deposition were utilized to produce these solid membranes. For low-temperature SOFC applications, G_0.2_DC thin films were fabricated on conductive Pt/ITO substrates. These were prepared using the EPD method with commercial and HY G_0.2_DC powders, followed by the SILAR-A+ process and post-hydrothermal annealing. The SILAR-A+ technique proved effective in producing thin, smooth solid membranes, although it is primarily efficient in oxidizing surface environments due to its successive ionic adsorption mechanism. To address this limitation, EPD on the Pt metal surface was conducted prior to SILAR-A+ deposition to form GDC thin films. Figure 6a shows SEM images of hybridized films produced through EPD (using commercial and HY G_0.2_DC powders) followed by the SILAR-A+ process and post-hydrothermal annealing. The surface morphology of films fabricated using EPD with HY G_0.2_DC and the SILAR-A+/hydrothermal method exhibited a much smoother and more homogeneous surface compared to other methods. The cross-sectional SEM images reveal the film thicknesses from the hybridization processes: 2.6 µm for EPD (commercial)/SILAR-A+-as-made, 1.5 µm for EPD (HY)/SILAR-A+-as-made, 1.2 µm after post-hydrothermal annealing of the EPD (commercial)/SILAR-A+ film, and 0.733 µm (733 nm) for the annealed EPD (HY)/SILAR-A+ film. Figure 6b presents the EIS and ionic conductivity measurements for Pt/G_0.2_DC/Pt-ITO films (1 cm × 1 cm).

The total conductivity (σ_Total_) values of the films with thicknesses of 1.2 µm and 733 nm were 2.68 × 10^−10^ S/cm and 1.39 × 10^−9^ S/cm at 300 °C, respectively (Figure S1 and Table S3). The successful fabrication of G_0.2_DC thin films using a hybrid approach combining EPD (HY GDC) and SILAR-A+ with post-hydrothermal annealing highlights the potential of these membranes as promising solid electrolyte candidates for low-temperature SOFC applications.

## 3. Experimental Procedure

The GDC powders were prepared using the solid-state (SS) method by mixing CeO_2_ (Alfa, 99.9%; Thermo Fisher Scientific Inc. Seoul, Republic of Korea) and Gd_2_O_3_ (Alfa, 99.9%), followed by annealing at 1400 °C for 3 h in air. Additionally, GDC powders were synthesized via the hydrothermal (HY) technique using precursors of 0.1 M Ce(CH_3_COO)_3_ (Alfa, 99.9%), 0.1 M Gd(NO_3_)_3_·6H_2_O (Alfa, 99.9%), and 0.1 M NaOH (Alfa, 98%) at 180 °C for 18 h. Phase identification of the synthesized powders was conducted using a Shimadzu XRD-6000 powder diffraction system (SHIMADZU CORPORATION, Kyoto, Japan) with Cu-Kα radiation. High-resolution synchrotron X-ray powder diffraction data were collected at the PLS-II 8D-XRS POSCO beamline of the Pohang Accelerator Laboratory (PAL). For the crystallization process, the hydrothermal synthesis was performed at 175 °C for 18 h. GDC thin films were fabricated on substrates using three methods: EPD, SILAR, and SILAR-A+. For the EPD process, 0.1 g of GDC powders (prepared by SS, HY, and commercial sources) was dispersed in 50 mL of acetone containing 0.02 g of iodine as an electrolyte. The dispersion underwent ultrasonication for 8 min, followed by electrophoretic deposition (EPD) using a DC power supply of 20 V for 15 s. For the SILAR method, 0.03 M solutions of Ce(CH_3_COO)_3_ Gd(NO_3_)_3_·6H_2_O, and NaOH were used as precursors. The substrate was attached to the arm of a Gilson 223 XYZ robotic sample changer for sequential adsorption (0.1 min), rinsing (0.1 min), and precipitation (0.1 min) reactions. Substrate surface pre-treatment involved 10 min of UVC exposure using an Omniscience UVC-150 system. The SILAR-A+ process incorporated an air-spraying step (0.1 min) at an air speed of 150 m/s as the final stage of the SILAR cycle. Thin film formation involved repeating the cycle 150 times in a sequence of cation adsorption, rinsing, anion reaction, rinsing, and air spraying. Hybridization of the EPD and SILAR-A+ methods was conducted on Pt/ITO substrates. The morphology of the GDC powders and films was characterized using scanning electron microscopy (SEM, Hitachi S-4200, Tokyo, Japan). The proportion of cracked versus crack-free areas in SEM images was quantified using the ImageJ (ver. 1.54k) program. Electrochemical impedance spectroscopy (EIS) was performed using a potentiostat (Ametek VERSASTAT3-500, AMETEK PAR(Princeton Applied Research), Oak, Ridge, TN, USA) over a frequency range of 1 MHz to 100 mHz. The ionic conductivity (σ) of GDC samples was calculated using the following equation:

σ = L/R·A

where σ, L, R, and A represent the ionic conductivity, the thickness of the GDC samples, the total resistance, and the contact area of the electrode with the samples, respectively [3,8].

## 4. Conclusions

Gd-doped CeO_2_ (GDC) nanoparticles were successfully prepared using three methods: hydrothermal synthesis, solid-state synthesis, and commercial sourcing. X-ray diffraction analysis and energy-dispersive X-ray spectroscopy were employed to characterize the F- and C-phase structures of CeO_2_/Gd_2_O_3_ system, along with the presence of oxygen vacancies. Thin, crack-free GDC films were fabricated using a combined approach of electrophoretic deposition (EPD) and the successive ionic layer adsorption and reaction-air spray plus (SILAR-A+) methods. Hydrothermal treatment further enhanced the film quality, as confirmed by SEM surface and cross-sectional imaging. Oxide-ion conductivities of the GDC pellets and thin films were measured using electrochemical impedance spectroscopy. The GDC pellets exhibited conductivity at 150 °C, while the thin films demonstrated conductivity at 300 °C. Additionally, G_0.2_DC thin films, approximately 730 nm thick, were successfully fabricated on Pt-ITO substrates, demonstrating their potential for use as solid electrolytes in low-temperature SOFC applications.

## Data Availability

The original contributions presented in this study are included in the article/Supplementary Materials. Further inquiries can be directed to the corresponding author(s).

## Supplementary Materials

The following supporting information can be downloaded at: https://www.mdpi.com/article/10.3390/molecules30030456/s1, Table S1. Cell parameters of Ce_1−*x*_Gd*_x_*O_2−*x*/2_ synthesized by hydrothermal and solid-stat methods and commercial G_0.2_DC; Table S2. Rietveld refinement and crystal data and refined atomic coordinates for Ce_0.8_Gd_0.2_O_1.9_ prepared by solid-state, hydrothermal, and commercial source; Figure S1. Ionic conductivities of Ce_1−*x*_Gd*_x_*O_2−*x*/2_ pellets prepared by HY, SS, and commercial sources at 150 °C and fitted via an equivalent circuit, and hybridizing G_0.2_DC thin-films at 300 °C; Table S3. Ionic conductivities of Ce_1−*x*_Gd*_x_*O_2−*x*/2_ pellets prepared by HY, SS, and commercial sources at 150 °C and fitted via an equivalent circuit, and hybridizing G_0.2_DC thin-films at 300 °C; Figure S2. SEM images of G_0.2_DC powders prepared by HY(A), SS(B), Commercial(C) sources.
