# Supplementary material for: Hybridizing Fabrications of Gd-CeO2 Thin Films Prepared by EPD and SILAR-A+ for Solid Electrolytes
Source: Molecules. 2025 Jan 21;30(3):456. doi: 10.3390/molecules30030456 (PMC11821242; doi:10.3390/molecules30030456)

Supporting Information

Hybridizing fabrications of Gd-CeO_2 thin-films prepared by EPD and SILAR-A+ for solid electrolytes

Taeyoon Kim[1] , Yun Bin Kim[1], Sungjun Yang[2] and Sangmoon Park*[1,3]

[1] Department of Engineering in Energy Materials, Graduate School, Silla University, Busan 46958, Republic of Korea
[2] UNIST Central Research Facilities, Ulsan National Institute of Science and Technology, Ulsan 44919, Republic of Korea
[3] Department of Environmental Energy & Chemistry, College of Engineering and Department of Fire Protection and Safety Management, College of Health and Welfare, Silla University, Busan 46958, Republic of Korea.

***Corresponding author:**

E-mail: spark@silla.ac.kr

Table S1. Cell parameters of $Ce_{1-x}Gd_xO_{2-x/2}$ synthesized by hydrothermal and solid-stat methods and commercial $G_{0.2}DC$.

**Hydrothermal** **$Ce_{1-x}Gd_xO_{2-x/2}$**	**a(Å)**	**V(Å^3)**	**R_p**	**Solid state** **$Ce_{1-x}Gd_xO_{2-x/2}$**	**a(Å)**	**V(Å^3)**	**R_p**
$x = 0$	5.4006(2)	157.52(1)	8.70	$x = 0$	5.39713(8)	157.214(2)	11.1
$x = 0.1$	5.4117(4)	158.49(1)	8.51	$x = 0.1$	5.40372(7)	157.790(2)	8.99
$x = 0.2$	5.4162(1)	158.88(2)	9.32	$x = 0.2$	5.41030(7)	158.367(3)	8.07
$x = 0.3$	5.4165(7)	158.91(1)	9.42	$x = 0.3$	5.41063(7)	158.396(3)	7.80
$x = 0.4$	5.4249(1)	159.65(4)	7.39	$x = 0.4$	5.41345(8)	158.644(3)	7.50
$x = 0.5$	5.4248(1)	159.64(4)	7.23	$x = 0.5$	5.41021(9)	158.539(3)	6.90
Commercial	5.4118(7)	158.50(3)	7.15				

Table S2. Rietveld refinement and crystal data and refined atomic coordinates for $Ce_{0.8}Gd_{0.2}O_{1.9}$ prepared by solid-state, hydrothermal, and commercial source.

Sample	$Ce_{0.8}Gd_{0.2}O_{1.9}$			
Synthesis method	Solid state		Hydrothermal	Commercial
Crystal system	Cubic		Cubic	Cubic
Space group	F m -3 m	I a -3	F m -3 m	F m -3 m
Lattice parameter (Å)	a = 5.40761 (4)	a = 10.85083 (9)	a = 5.426 (2)	a = 5.421 (3)
Volume (Å^3)	158.130 (1)	1277.582 (12)	V = 159.767 (16)	V = 159.349 (26)
Fraction (%)	47.55 (52)	52.45 (57)	100	100
R_p	1.60		4.39	4.44
R_{wp}	2.59		5.46	5.53
R_{exp}	2.61		2.03	1.71
S	0.99		2.7	3.2
χ^2	0.979		7.16	10.5

Solid State method						
Atom	Wyckoff Position	x	y	z	B_{iso}	SOF
F m -3 m						
Ce	4a	0.000	0.000	0.000	0.9 (2)	0.8
Gd	4a	0.250	0.000	0.250	0.9 (2)	0.2
O2	16c	0.250	0.250	0.250	0.9	0.95
I a -3						
Ce1	24d	0.2397 (3)	0.000	0.250	0.8 (2)	0.8
Gd1	24d	0.2397 (3)	0.000	0.250	0.8 (2)	0.2
Ce2	8a	0.000	0.000	0.000	0.7 (6)	0.8
Gd2	8a	0.000	0.000	0.000	0.7 (6)	0.2
O1	48e	0.125	0.375	0.125	1.8	1.0
O2	16c	0.125	0.125	0.125	1.8	1.0
Hydrothermal method						
Atom	Wyckoff Position	x	y	z	B_{iso}	SOF
F m -3 m						
Ce	4a	0.000	0.000	0.000	0.9 (2)	0.8
Gd	4a	0.250	0.000	0.250	0.9 (2)	0.2
O2	16c	0.250	0.250	0.250	0.9	0.95
Commercial						
Atom	Wyckoff Position	x	Y	z	B_{iso}	SOF
F m -3 m						
Ce	4a	0.250	0.000	0.250	0.55 (13)	0.8
Gd	4a	0.250	0.000	0.250	0.55 (13)	0.2
O2	16c	0.125	0.125	0.125	0.56	0.95

Figure S1. Ionic conductivities of $Ce_{1-x}Gd_xO_{2-x/2}$ pellets prepared by HY, SS, and commercial sources at 150 °C and fitted via an equivalent circuit, and hybridizing $G_{0.2}DC$ thin-films at 300 °C.

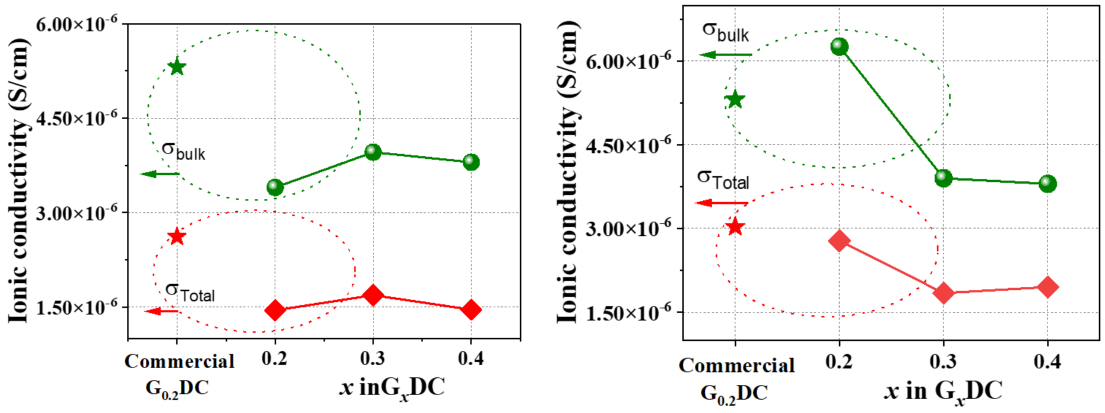

Hydrothermal Solid State

Table S3. Ionic conductivities of $Ce_{1-x}Gd_xO_{2-x/2}$ pellets prepared by HY, SS, and commercial sources at 150 °C and fitted via an equivalent circuit, and hybridizing $G_{0.2}DC$ thin-films at 300 °C.

**Hydrothermal $Ce_{1-x}Gd_xO_{2-x/2}$**	**R_{bulk} (kΩ)**	**R_{Total} (kΩ)**	**σ_{bulk} (S/cm) at 150°C**	**σ_{Total} (S/cm) at 150°C**
x =0.2	19.3	45.7	$3.4x10^{-6}$	$1.4x10^{-6}$
x =0.3	16.6	38.9	$3.9x10^{-6}$	$1.7x10^{-6}$
x =0.4	16.1	39.9	$3.8x10^{-6}$	$1.4x10^{-6}$
**Solid state $Ce_{1-x}Gd_xO_{2-x/2}$**	**R_{bulk} (kΩ)**	**R_{Total} (kΩ)**	**σ_{bulk} (S/cm) at 150°C**	**σ_{Total} (S/cm) at 150°C**
x =0.2	12.4	27.9	$6.2x10^{-6}$	$2.8x10^{-6}$
x =0.3	19.8	41.9	$3.9x10^{-6}$	$1.8x10^{-6}$
x =0.4	16.1	39.9	$3.8\ x10^{-6}$	$1.9x10^{-6}$
**Commercial $G_{0.2}DC$**	14.4	25.0	$5.3x10^{-5}$	$3.1x10^{-6}$

Ionic conductivity data of $G_{0.2}DC$ samples fitted via an equivalent circuit using the EIS Spectrum Analyzer software.

$G_{0.2}DC$	R_{Bulk} (kΩ)	R_{GB} (kΩ)	R_{Total} (kΩ)	σ_{bulk} (S/cm) at 150°C	σ_{GB} (S/cm) at 150°C	σ_{Total} (S/cm) at 150°C
**Commercial**	**13.8**	**11.4**	**25.3**	**5.4×10^{-6}**	**6.6×10^{-6}**	**2.9×10^{-6}**
**Hydrothermal**	**18.9**	**26.3**	**45.2**	**4.0×10^{-6}**	**2.9×10^{-6}**	**1.6×10^{-6}**
**Solid state**	**12.0**	**16.7**	**28.7**	**6.3×10^{-6}**	**4.5×10^{-6}**	**2.6×10^{-6}**

**Hybridizing $G_{0.2}DC$ Thin Films**	**L (μm)**	**σ (S/cm) at 300°C**
EPD (Commercial) / SILAR-A+	1.2μm	2.68×10^{-10} S/cm
EPD (Hydrothermal) / SILAR-A+	0.73μm	1.39×10^{-9} S/cm

Figure S2. SEM images of $G_{0.2}DC$ powders prepared by HY(A), SS(B), Commercial(C) sources.

A. Hydrothermal B. Solid-state C. Commercial

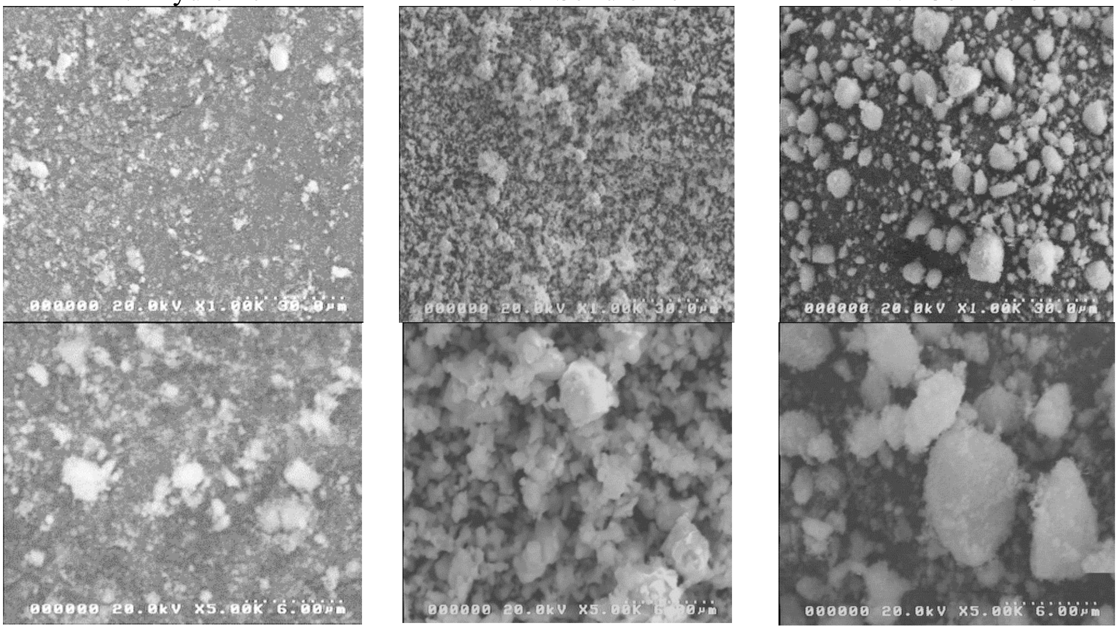

Supplement: Supplementary file 1 [file molecules-30-00456-s001.zip › molecules-3397343-supplementary.pdf]
